# The Vaccine Efficacy Against the SARS-CoV-2 Omicron: A Systemic Review and Meta-Analysis

**DOI:** 10.3389/fpubh.2022.940956

**Published:** 2022-07-13

**Authors:** Yuntao Zou, Doudou Huang, Qian Jiang, Yanglin Guo, Chider Chen

**Affiliations:** ^1^Department of Oral and Maxillofacial Surgery and Pharmacology, School of Dental Medicine, University of Pennsylvania, Philadelphia, PA, United States; ^2^Department of Medicine, Saint Peter's University Hospital, New Brunswick, NJ, United States; ^3^Medical School of Nanjing University, Nanjing, China; ^4^Department of Orthodontics, Nanjing Stomatological Hospital, Medical School of Nanjing University, Nanjing, China; ^5^Division of Pulmonary, Critical Care, and Sleep Medicine, University of Mississippi Medical Center, Jackson, MS, United States; ^6^Center of Innovation and Precision Dentistry, School of Dental Medicine, School of Engineering and Applied Sciences, University of Pennsylvania, Philadelphia, PA, United States

**Keywords:** booster, infection, meta-analysis, Omicron variant, vaccination

## Abstract

**Background:**

COVID-19 is a respiratory illness caused by SARS-CoV-2. The most recent variant is Omicron (line B.1.1.529), which was first identified in South Africa in November 2021. The concern with this variant is the ineffectiveness of vaccines currently available. We aim to systematically evaluate the effectiveness of the currently available COVID-19 vaccines and boosters for the Omicron variant.

**Methods:**

We searched the PubMed, Embase, the Cochrane Library and Web of Science databases from inception to June 5th, 2022. Studies that examined the effectiveness of SARS-CoV-2 vaccines against the Omicron variant infection were included. Random-effects model was used to estimate the pooled vaccine effectiveness against the Omicron variant.

**Results:**

A total of 13 studies were included to evaluate the effectiveness of the vaccine against the Omicron variant, and 11 studies were included to compare the effectiveness between the two-dose and three-dose (booster) vaccinations. Full vaccination (two-dose with or without booster) showed a protective effect against the Omicron variant compared to no vaccination (OR = 0.62, 95% CI: 0.56–0.69), while the effectiveness decreased significantly over 6 months after the last dose. The two-dose vaccination plus booster provided better protection against the Omicron variant compared to the two-dose vaccination without booster (OR = 0.60, 95% CI: 0.52–0.68). Additional analysis was performed for the most commonly used vaccines in the United Staes: BNT162b2(Pfizer) (OR = 0.65, 95% CI: 0.52–0.82) and mRNA-1273(Moderna) (OR = 0.67, 95% CI: 0.58–0.88) vaccines in the US, which showed similar effectiveness compared to no vaccination.

**Conclusions:**

The full dose of SARS-CoV-2 vaccination effectively reduces infection from the SARS-CoV-2 Omicron variant; however, the effectiveness wanes over time. The booster vaccine provides additional protection against the Omicron variant.

## Introduction

Coronavirus disease 2019 (COVID-19) is caused by Severe Acute Respiratory Syndrome Coronavirus 2 (SARS-CoV-2) (1). Since the first reported case in December 2019, in Wuhan, China, the virus has spread through China and many other countries worldwide in a short period of time (2). The COVID-19 pandemic has changed the lives of billions of people all over the world and has significantly weakened the global economy. Approximately 400 million people worldwide have been infected by this virus, resulting in around 6 million deaths (3). Consequently, there is an urgent need and of great importance to prevent COVID-19 infections with the implementation of a safe and effective vaccination program.

International researches proceeded at an unprecedented speed in the pursuit of an effective and safe vaccine against COVID-19. In this regards, a variety of different types of vaccines is currently being administered to people of various ages all around the world (4, 5). It is commonly recognized that the mRNA vaccine is the most effective type of vaccine, followed by the viral vector vaccines and inactivated virus vaccines (6, 7). Nonetheless, all vaccines are effective tools in preventing severe COVID-19 symptoms, hospitalization, and death (4).

COVID-19 has become increasingly virulent due to the emergence of variants (8, 9). Recently, a new variant of SARS-CoV-2 was reported from South Africa. On November 26th, 2021, the World Health Organization (WHO) designated this mutant as a variant of concern—Omicron (line B.1.1.529) (10). The B.1.1.529 Omicron variant has enhanced transmissibility and immune evasion, and has been observed in over 77 countries (11). This raises a concern regarding the efficacy of existing COVID-19 vaccines against the Omicron variant (12), and also stimulates the debate for a booster vaccine (13). Our study aims to provide a comprehensive understanding of the effectiveness of the existing COVID-19 vaccines and whether the booster provides additional protection against the B.1.1.529 Omicron variant. Clinically, our findings will help to develop vaccination strategies against the Omicron variant.

## Methods

### Study Design That Contains Inclusion and Exclusion Criteria

Human studies were included and studies with specifically targeted participants (e.g., patients with severe systemic diseases, patients with prolonged antibiotic therapy, pregnant women, cancer patients, frontline workers, nursing home employees) were excluded. Studies focused on the effectiveness of COVID-19 vaccines against the Omicron variant, and the effectiveness on vaccinated participants vs. unvaccinated participants were included. No restrictions were applied to the age of participants, the types of vaccination, or the number of participants.

### Search Strategy

A comprehensive search was conducted for academic research studies that reported the effectiveness of COVID-19 vaccines against the B.1.1.529 Omicron variant published from inception to June 5th, 2022 by searching the following electronic bibliographic databases: PubMed, Embase, the Cochrane Library, and Web of Science. The following search terms were used: (“Covid19 variant” OR “Omicron” OR “B.1.1.529” OR “BA lineages” OR “21H variant” OR “BA.2” OR “21K variant”) AND (“Vaccine” OR “vaccination” OR “BNT162b2” OR “Pfizer” OR “BNT-162C2” OR “Tozinameran” OR “Comirnaty” OR “mRNA-1273” OR “Moderna” OR “Elasomeran” OR “TAK-919” OR “M-1273”).

### Data Collection and Analysis

Two independent investigators (Y.Z., D.H.) assessed the articles and extracted data according to the inclusion and exclusion criteria. These two independent investigators (Y.Z., D.H.) also assessed the methodological quality of the trials included in this review. A Microsoft Excel database was created to record all available information, including vaccine type, doses, and vaccination rate in infection and control groups. Discrepancies were discussed and resolved by consensus. The Newcastle-Ottawa assessment scale was used to assess the quality of evidence.

### Statistical Analysis

Stata version 16.0 (Stata corp., college station, TX, USA) was used to perform the meta-analyses. Pooled odds ratio (OR) with 95% confidence intervals (CI) were calculated by the random-effects model to accommodate heterogeneity across studies. Heterogeneity was assessed by I^2^ statistics.

## Results

### Description of Included Studies

**Figure 1:**
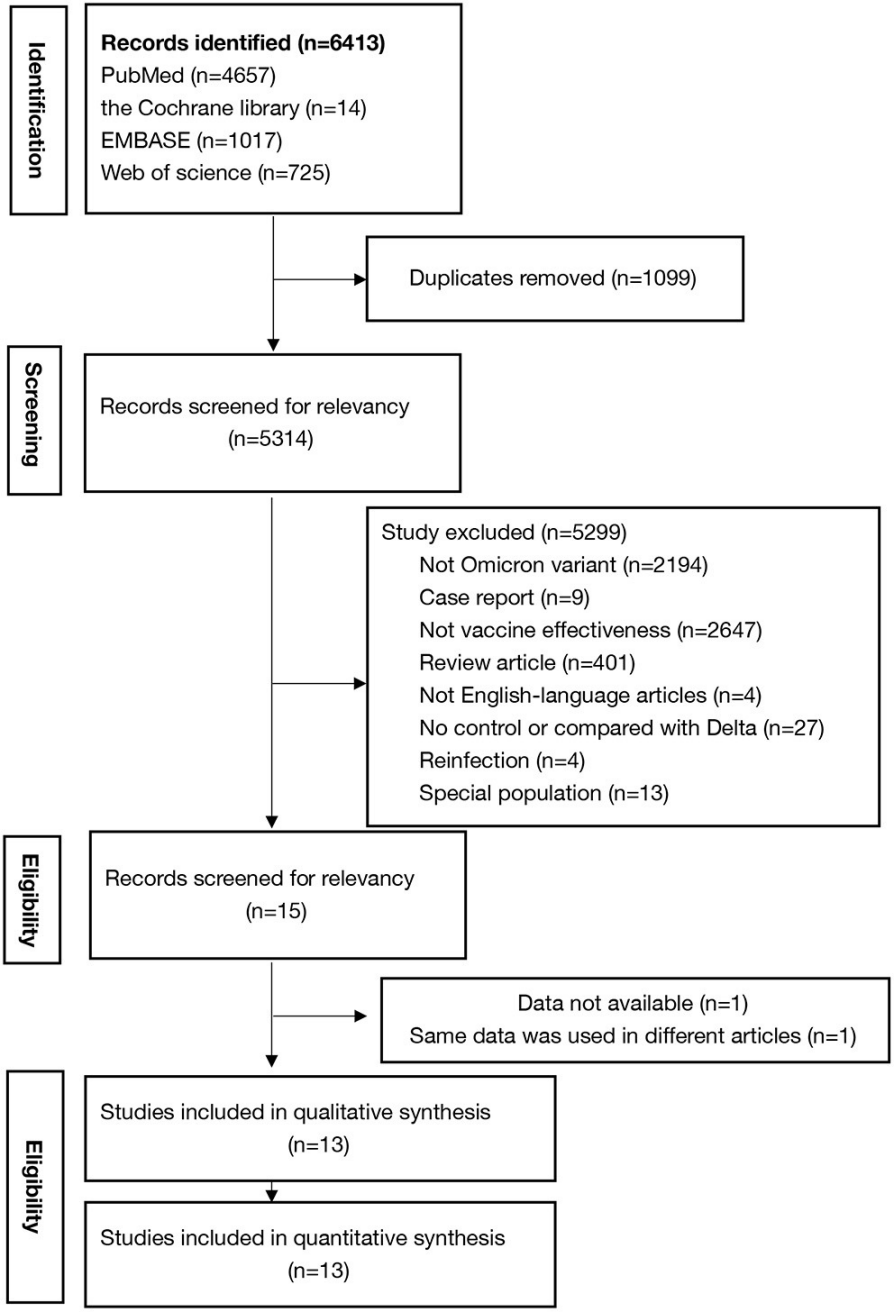
Steps of the study selection procedures.

The search of databases yielded 6,413 results, of which 13 studies (12, 14–25) met the criteria and were included in this study. Andrews et al. (26) also reported effectiveness of COVID-19 vaccines against the Omicron variant in 2021, but the included cases could be reported in his another article published in 2022 according to the timetable and the inclusion criteria. Figure 1 shows the detail selection procedures to identify the included studies. Among them, five included studies have low risk of bias and eight have high risk of bias (Supplementary Table 1). The included studies had a total sample of around one hundred million (N = 148260342) participants and of these, 74.2% were fully vaccinated (*n* = 110076947) with at least two-doses of COVID-19 vaccines (Table 1). Among the 13 studies, 1 study reported the effectiveness of the BNT162b2 (Pfizer–BioNTech), ChAdOx1 (AstraZeneca), mRNA-1273 (Moderna) and JNJ-78436735 vaccines (25); 1 study reported the effectiveness of the BNT162b2 and ChAdOx1 vaccines (24); 3 studies reported the effectiveness of the BNT162b2, ChAdOx1, and mRNA-1273 vaccines (12, 21, 22); 5 studies reported the effectiveness of the BNT162b2, and mRNA-1273 vaccines (14, 16, 17, 20, 23); 2 studies reported the effectiveness of the BNT162b2 vaccine (18, 19), and 1 study reported the effectiveness of the mRNA-1273 vaccine (15).

**Table 1:** Characteristics of the selected studies.

**Study**	**Country**	**Study design**	**Vaccine type**	**Positive**		**Negative**	
				Fully vaccinated^*^	**Not vaccinated**	Fully vaccinated^*^	**Not vaccinated**
Lauring et al. (14)	USA	Case-control	BNT162b2, mRNA-1273	291	268	3908	2054
Tseng et al. (15)	USA	Case-control	mRNA-1273	13412	8590	32882	17051
Accorsi et al. (16)	USA	Case-control	BNT162b2, mRNA-1273	9686	3412	38043	8721
Thompson et al. (17)	USA	Case-control	BNT162b2, mRNA-1273	3272	3572	8876	3884
Collie et al. (18)	South Africa	Case-control	BNT162b2	9700	7889	35957	18442
Andrews et al. (12)	UK	Case-control	ChAdOx1, BNT162b2, mRNA-1273	753437	101109	1285532	107238
Klein et al. (19)	USA	Case-control	BNT162b2	1050	4434	1527	5203
Ferdinands et al. (20)	USA	Case-control	BNT162b2, mRNA-1273	10289	13991	20464	10808
Kodera et al. (23)	Japan	observatory	BNT162b2, mRNA-1273	25187	12681	103065994	35329428
Acuti Martellucci et al. (25)	Italy	Cohort	ChAdOx1, BNT162b2, mRNA-1273, JNJ-78436735	95714	41281	827293	252421
Horne et al. (24)	UK	Cohort	ChAdOx1, BNT162b2	845048	90451	1858123	2033092
Kirsebom et al. (22)	UK	Case-control	ChAdOx1, BNT162b2, mRNA-1273	437276	59793	561848	37280
Sheikh et al. (21)	UK	Cohort	ChAdOx1, BNT162b2, mRNA-1273	12067	1003	120071	9299
Total	*n* = 13			2216429	348474	107860518	37834921

* *Fully vaccinated group included people had received at least two vaccine doses*.

### Effectiveness of the COVID-19 Vaccines Against the Omicron Variant

**Figure 2:**
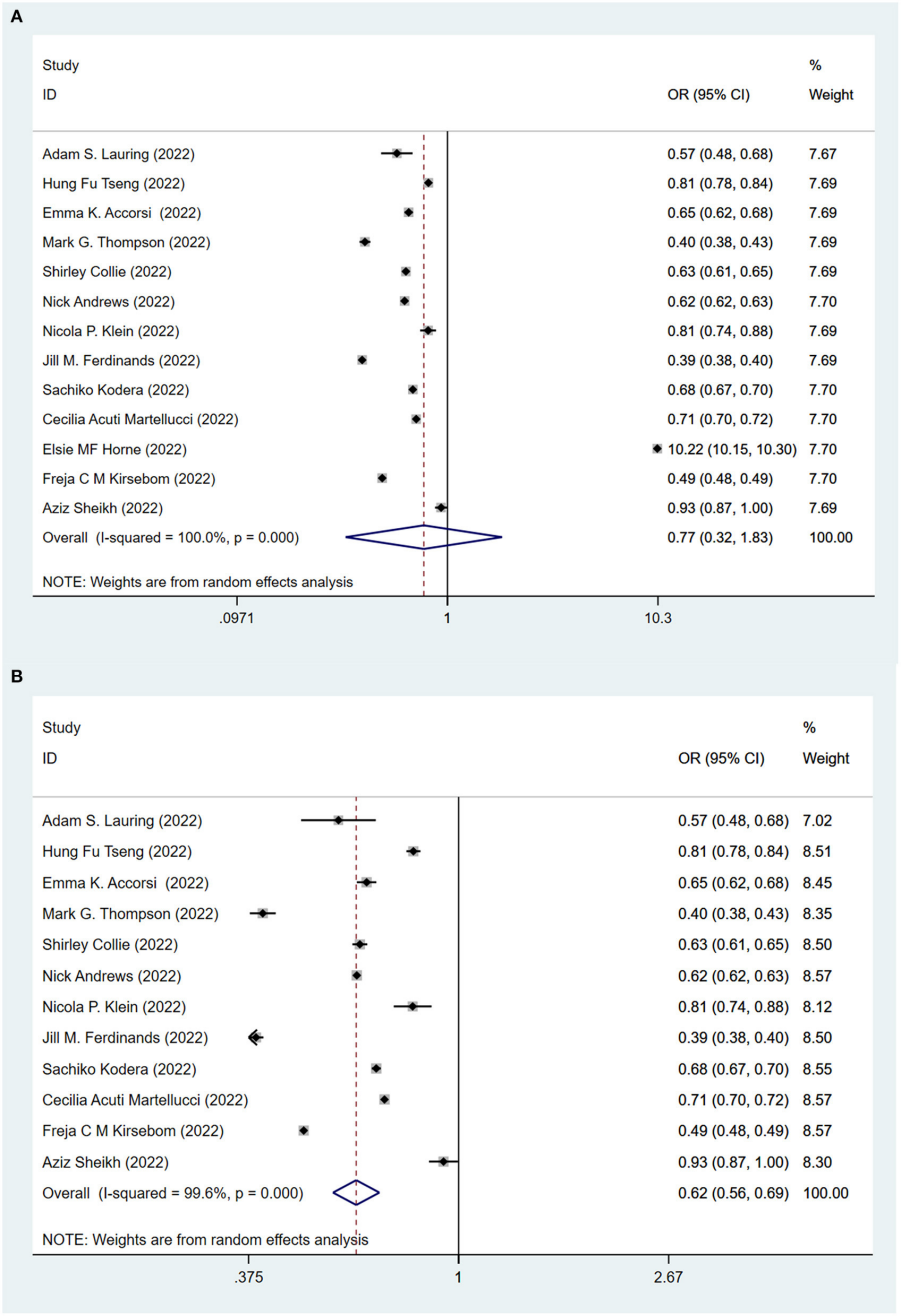
Effectiveness of the fully COVID-19 vaccines (at least two doses) against the Omicron variant compared to the unvaccination. **(A)** 13 studies included. **(B)** 12 studies included and excluded one study about effectiveness of vaccines over 6 months since second dose.

To evaluate the protective role of COVID-19 vaccination, we first compare fully vaccinated (at least two standard doses) and unvaccinated populations. Figure 2A shows the effectiveness of the COVID-19 vaccines against the Omicron variant after at least two doses of the vaccination. All (12, 14–23, 25) but one study (24) showed that vaccines played a significant role in reducing the risk of B.1.1.529 Omicron variant infection among the fully vaccinated population (two doses with or without boosters), compared to the unvaccinated population. The pooling of 13 studies showed that no significant difference between fully vaccinated population and unvaccinated population (OR = 0.77, 95% CI: 0.32–1.83). After excluding the one study (24) looking at the long term effectiveness of the vaccinated population, the pooling of the remaining 12 studies showed that full vaccination significantly lowered the risk (OR = 0.62, 95% CI: 0.56–0.69) of infection against the Omicron variant (Figure 2B).

**Figure 3:**
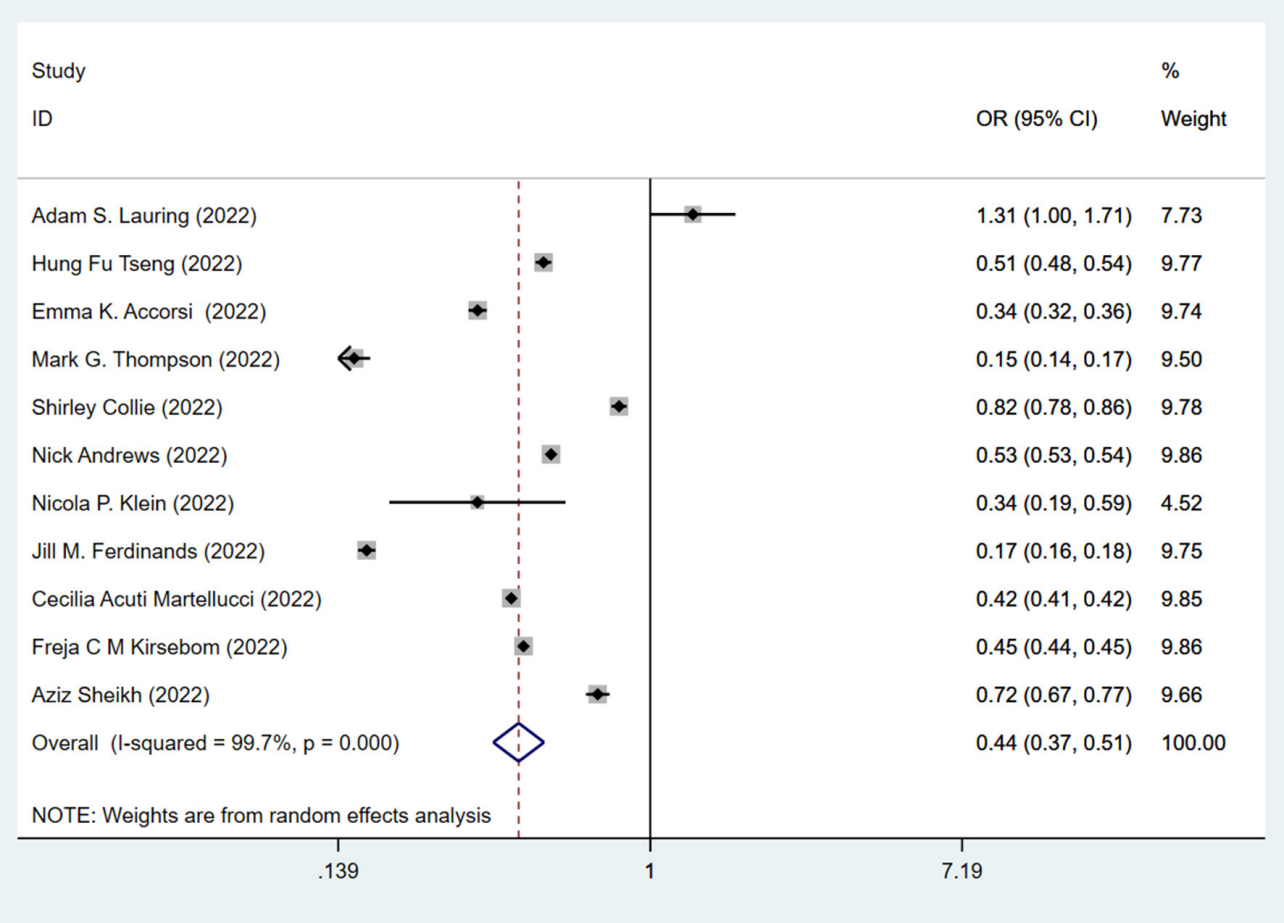
Effectiveness of the three doses COVID-19 vaccines against the Omicron variant compared to the unvaccination.

**Figure 4:**
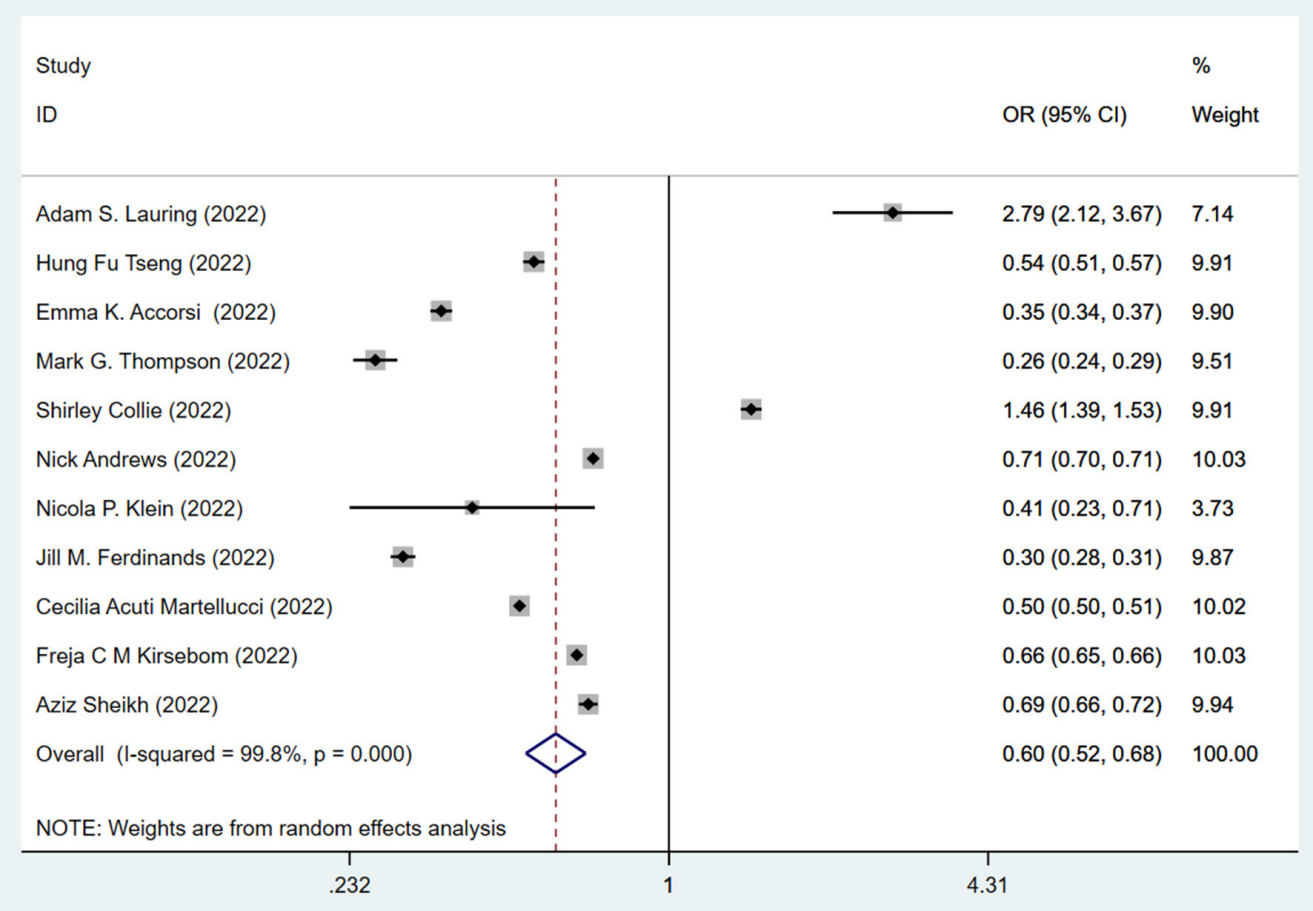
Effectiveness of the three doses COVID-19 vaccines against the Omicron variant compared to the two doses only vaccination.

This data prompted us to examine the effectiveness of the booster vaccination. The effectiveness of the standard two-dose vaccination plus one booster (total three doses) against the Omicron variant is shown in Figure 3. The pooling of 11 studies showed that two-dose vaccination plus booster significantly lowered the risk (OR = 0.44, 95% CI: 0.37–0.51; I^2^ = 99.7%, *p* < 0.001) of infection against the Omicron variant compared to the unvaccinated group (Figure 3). The pooling of these same studies also showed that the standard two-dose vaccination plus a booster significantly lowered the risk (OR = 0.60, 95% CI: 0.52–0.68) of infection against the Omicron variant compared to the two-dose vaccination without booster (Figure 4), with a significant heterogeneity (I^2^ = 99.8%, *p* < 0.001). Collectively, these analyses reveal that full vaccination builds a protective effect against the Omicron variant, and booster vaccines provide additional protection against the Omicron variant.

**Figure 5:**
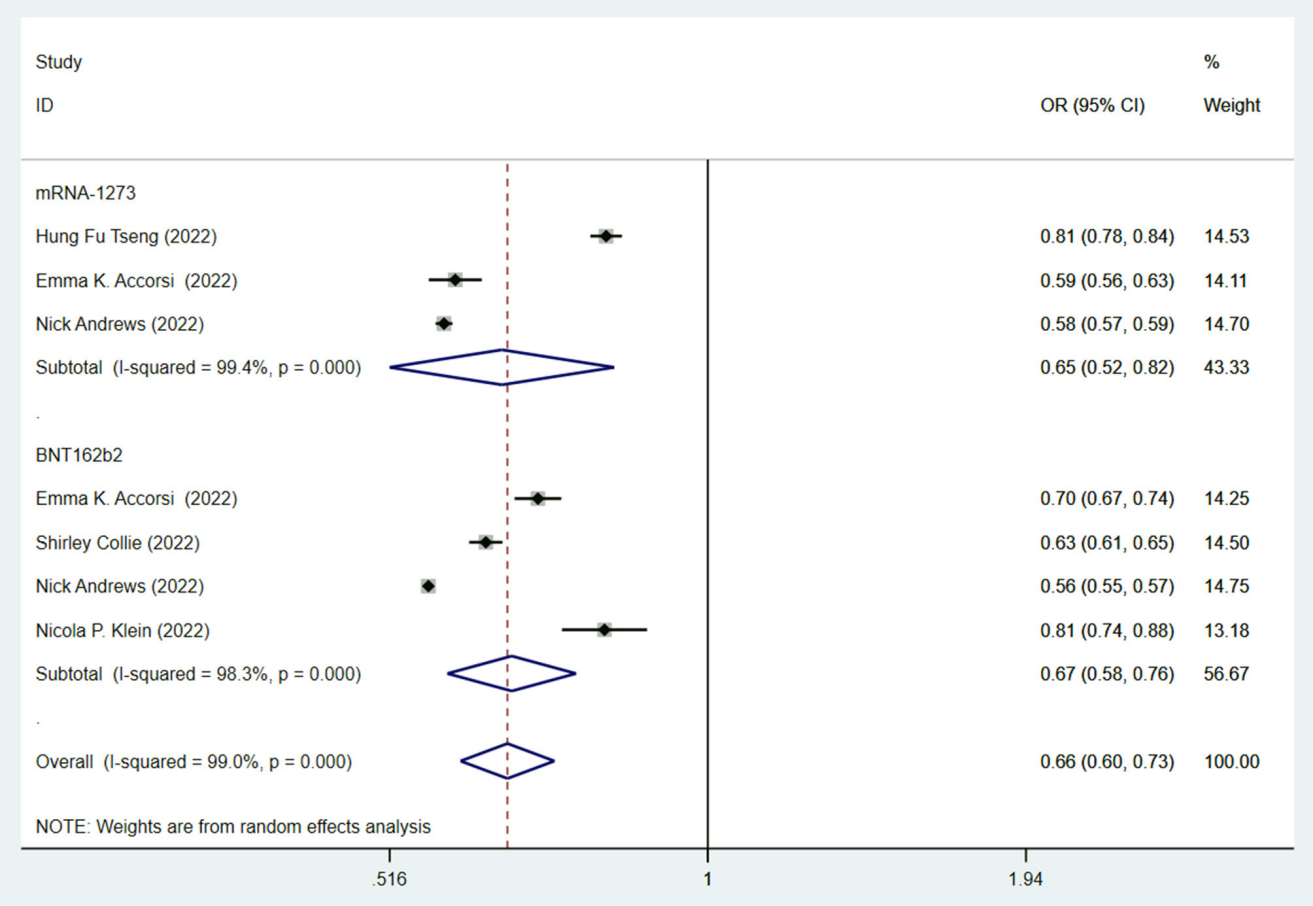
Effectiveness of fully mRNA vaccines (BNT162b2 and mRNA-1273) against the Omicron variant compared to the unvaccination.

Since mRNA vaccines were commonly recognized as the most effective vaccines (6, 7), we then studied the efficacy of mRNA vaccines against the Omicron variant. Both the BNT162b2 (OR = 0.65, 95% CI: 0.52–0.82; I2 = 99.4%, *p* < 0.001) and mRNA-1273 (OR = 0.67, 95% CI: 0.58–0.88; I2 = 98.3%, *p* < 0.001) vaccines have similar effectiveness and significantly lowered the risk of infection against the Omicron variant compared to no vaccination (Figure 5).

In summary, our data clearly indicated the effectiveness of vaccination and the promoter role of booster vaccination against the Omicron variant. The results also confirmed the effectiveness of the mRNA vaccines in preventing the Omicron variant infections.

## Discussion

The recent emergence of the SARS-CoV-2 Omicron variant has attracted worldwide attention and became a global concern (11). Among the different variants of SARS-CoV-2, the Omicron variant has a remarkably increased transmissibility (27, 28). It is also difficult to evaluate the effectiveness of the currently available COVID19 vaccines against the Omicron variant in the absence of strong data (29). Besides, there are a lot of debates about the necessity of the boosters, and the new emerging Omicron variant with the significantly increased transmissibility supercharges the debates (13). The goal of this study is to investigate the roles of COVID-19 vaccination in preventing the Omicron variant infections. The results showed the effectiveness of full vaccination against the Omicron variant was significantly but waned over time. The booster dose could induce higher levels of effectiveness compared to the standard two-dose vaccination, which may be related to the shorter length of time between the last dose and the study data collection since the vaccination effectiveness wanes over time. These findings will facilitate vaccination strategies and help develop booster schedules.

Full vaccination with the currently available vaccines provides a statistically significant protection against the Omicron variant (OR = 0.62, 95% CI: 0.56–0.69) within a period of time, but it is not as effective as it is against the Delta variant (RR = 0.15, 95% CI: 0.07–0.31) (30). The booster provides additional protection against Omicron (OR = 0.44, 95% CI: 0.37–0.51); however, it is also less effective compared to the reported protection effectiveness against Delta (OR = 0.065, 95% CI: 0.059–0.071) (16). It is expected that new variants will continue to emerge as viruses mutates. Although the effectiveness of the currently available vaccines against new variants may decrease, vaccination still provides protection against severe COVID-19 caused by different variants (31, 32) and may also decrease the emergence of new variants.

While Omicron has been less virulent than the Alpha and Delta variants, which was likely related to higher vaccination rates and thus reduced disease severity (33), it has still caused tremendous morbidity and mortality (14). In the absence of an Omicron variant-specific vaccines, the currently existing vaccines remain the best option to reduce infection and disease severity against the currently circulating SARS-CoV-2 Omicron variant. The booster is also recommended given the increased transmissibility of the Omicron variant and reduced protective effectiveness of the standard two-dose vaccination compared to the three-dose vaccination with booster, especially for the high-risk populations. Additionally, those who received the two-dose vaccination plus booster were also reported to have fewer hospitalizations and lower disease severity compared to the unvaccinated population (14, 15).

Several COVID-19 vaccines (BNT162b2, ChAdOx1, mRNA-1273, and JNJ-78436735) were included in this analysis since the availability of different types of vaccinations were varied across the world. Different vaccines may have various protective effects again COVID-19 variants. An additional analysis was performed to assess the effectiveness of mRNA vaccines (BNT162b2 and mRNA-1273), which are recognized as the most effective (6, 7) against the Omicron variant. Although the results showed both the BNT162b2 and mRNA-1273 vaccines significantly lowered the risk of infection against the Omicron variant, interpretation of the results must consider the small number of included studies (3 for mRNA-1273 and 4 for BNT162b2). These studies did not evaluate the time between the second or third vaccinated doses and symptom onset, which might influence the results as the vaccine effectiveness decreased with time (15).

In this analysis, the effectiveness of the vector vaccine (ChAdOx1) was reported by in five studies (12, 21, 22, 24, 25). The pooled effectiveness of the vector vaccine was unable to be evaluated as most of these studies did not report the effectiveness of each vaccine individually. The ChAdOx1 vector vaccine followed by boosting with a mRNA vaccine (BNT162b2 or mRNA-1273) is currently recommended in Germany. This vaccination strategy is reported to induce higher or comparable humoral and cellular immune responses than homologous mRNA vaccines (34). More studies are needed in the future to assess the effectiveness of a specific vaccine and vaccination strategy. Moreover, the misclassification of disease status could be a potential source of bias. Several studies (12, 15, 18, 21, 22, 25) used highly specific and sensitive PT-PCR tests, which minimized misclassification and is recommended for future studies in this field.

Except for the vaccinated doses, type and time, the intrinsic host factors (age, sex, genetics, comorbidities and so on), and extrinsic factors (preexisting immunity, microbiota, infections, antibiotics and so on) also influence humoral and cellular vaccine responses in humans (35). Besides, vaccine adjuvant and administration factors (schedule, site, route, and coadministered vaccines and other drugs) are also influential (35). Additional research will provide more information about these factors to help create effective vaccination strategies.

## Data Availability Statement

The raw data supporting the conclusions of this article will be made available by the authors, without undue reservation.

## Author Contributions

Study design: CC and QJ. Data acquisition and analysis of data: YZ and DH. Manuscript drafting and manuscript editing: YZ, DH, QJ, YG, and CC. All authors contributed to the article and approved the submitted version.

## Funding

This work was supported by grants from National Institute of Dental and Craniofacial Research, National Institutes of Health, Department of Health and Human Services (R00DE025915 and R03DE028026 to CC).

## Conflict of Interest

The authors declare that the research was conducted in the absence of any commercial or financial relationships that could be construed as a potential conflict of interest.

## Publisher's Note

All claims expressed in this article are solely those of the authors and do not necessarily represent those of their affiliated organizations, or those of the publisher, the editors and the reviewers. Any product that may be evaluated in this article, or claim that may be made by its manufacturer, is not guaranteed or endorsed by the publisher.
