# Supplementary material for: The Vaccine Efficacy Against the SARS-CoV-2 Omicron: A Systemic Review and Meta-Analysis
Source: Front Public Health. 2022 Jul 13;10:940956. doi: 10.3389/fpubh.2022.940956 (PMC9326247; doi:10.3389/fpubh.2022.940956)
Supplement: Supplementary file 1 [file Table_1.DOCX]

Supplementary Material

Supplemental Table. Newcastle-Ottawa assessment scale for case-control studies.

| Study | Selection | | | | Comparability | Outcome | | | Score  （risk of bias） |
| --- | --- | --- | --- | --- | --- | --- | --- | --- | --- |
|  | 1 | 2 | 3 | 4 | 1 | 1 | 2 | 3 |  |
| Adam S. Lauring (2022) | a | a | b | b | a | c | a | a | 5 |
| Hung Fu Tseng (2022) | a | a | c | a | a | a | a | a | 7 |
| Emma K. Accorsi (2022) | a | a | a | a | a | a | a | a | 8 |
| Mark G. Thompson (2022) | a | a | b | b | a | a | a | a | 6 |
| Shirley Collie (2022) | a | a | b | b | a | a | a | a | 6 |
| Nick Andrews (2022) | a | a | a | b | a | a | a | a | 7 |
| Nicola P. Klein(2022) | a | a | b | b | a | e | a | a | 5 |
| Jill M. Ferdinands(2022) | a | a | b | b | a | a | a | a | 6 |
| Sachiko Kodera(2022) | a | a | c | b | a | a | a | a | 6 |
| Cecilia Acuti Martellucci(2022) | a | a | a | b | a | a | a | a | 7 |
| Elsie MF Horne(2022) | a | a | c | b | a | a | a | a | 6 |
| Freja C M Kirsebom(2022) | a | a | c | b | a | a | a | a | 6 |
| Aziz Sheikh(2022) | a | a | a | b | a | a | a | a | 7 |

**Selection**

(1) Is the case definition adequate? a: yes, with independent validation*; b: yes, eg record linkage or based on self-reports; c: no description

(2) Representativeness of the cases. a: consecutive or obviously representative series of cases*; b: potential for selection biases or not stated

(3) Selection of Controls. a: community controls*; b: hospital controls; c: no description

(4) Definition of Controls. a: no history of disease (endpoint) *; b: no description of source

**Comparability**

(1) Comparability of cases and controls on the basis of the design or analysis. a: study controls for the most important factor*; b. study controls for any additional factor*

**Exposure**

(1) Ascertainment of exposure. a: secure record*; b: structured interview where blind to case/control status*; c: interview not blinded to case/control status; d: written self-report or medical record only; e: no description

(2) Same method of ascertainment for cases and controls. a: yes*; b: no

(3) Non-Response rate. a: same rate for both groups*; b: non-respondents described; c: rate different and no designation

The score is the total number of stars. A study with score from 7-8 has low risk, 4-6 has high risk, and 0-3 has very high risk of bias.
